# Supplementary material for: Efficacy of abiraterone acetate for high-risk hormone-naïve metastatic prostate cancer: A comparison with combined androgen blockade therapy with bicalutamide and androgen deprivation therapy alone
Source: PLoS One. 2022 Oct 20;17(10):e0276081. doi: 10.1371/journal.pone.0276081 (PMC9584391; doi:10.1371/journal.pone.0276081)
Supplement: Attachment — Submitted filename: Response to Reviewers.docx [file pone.0276081.s002.docx]

Point-by-point responses

We would like to thank the editors and reviewer for helpful and insightful comments. We agreed with the points raised by them and revised the manuscript.

**Reviewer #1 comment 1**:

The authors evaluated the treatment efficacy of abiraterone for the management of hormone naïve metastatic prostate cancer. The main outcomes were time to castration resistance (TTCR) and PSA response. Slight typographical errors in Lines 171-173 and 226.

**Response**:

We thank the reviewer for noting these typographical errors. We have corrected these typographical errors.

**Reviewer #1 comment 2**:

By showing a prolonged TTCR in the ABI group, the authors have concluded that abiraterone is superior to other treatments. Without discussing the tolerability of abiraterone, the data only suggests a superiority in efficacy. For clarity, the authors should specify "superiority in treatment efficacy".

**Response**:

We thank the reviewer for raising an important point. We reviewed the medical records of all patients and investigated adverse events. Adverse events with dose reduction or interruption occurred in 7 patients, all in the abiraterone group. Most of them were ALT/AST increase. All patients who interrupted dose resumed abiraterone and none discontinued.

We add the Table 3 which shows details of the patients in the results section and add “Adverse events with dose reduction or interruption occurred in 7 patients, all in the abiraterone group. Most of them were ALT/AST increase. All patients who interrupted dose resumed abiraterone and none discontinued. Details of adverse events of abiraterone group were shown in Table 3.” and add sentences in the discussion section as follows: “In this study, adverse events with dose reduction or interruption occurred in 7 patients, all in the abiraterone group. However, all patients who interrupted dose resumed abiraterone and none discontinued. Therefore, men with high-risk mHNPC treated with abiraterone had a manageable safety profile.”

**Reviewer #1 comment 3**:

The duration of follow-up is unclear. Were any patients lost to follow-up? Were all patients followed-up uniformly for the same duration?

**Response**:

As described in the results section, the median (range) follow-up period in the ABI, CAB, and ADT groups was 12.6 (3–90), 9.6 (1–115), and 9.6 (3–52) months, respectively. In total, 9, 69, and 27 men in the ABI, CAB, and ADT groups, respectively, progressed to castration-resistant prostate cancer (CRPC) during the follow-up period.

We did not mention, but 13 patients were referred to another hospital during the treatment, 6 in the ABI and 7 in the CAB groups, and 3 patients died of other causes. The median follow-up period of ABI group was a little longer than CAB and ADT groups, but we followed these patients uniformly though time of treatment initiation varied.

We add a sentence in the results section as follows: “The median follow-up period of ABI group was a little longer than CAB and ADT groups, but we followed these patients uniformly though time of treatment initiation varied. Thirteen patients were referred to another hospital during the treatment, 6 in the ABI and 7 in the CAB groups, and 3 patients died of other causes.”

**Reviewer #1 comment 4**:

The clinicopathologic data shows that the groups were fairly comparable. Given the limited sample size, was the baseline testosterone level comparable between groups? If the pre-treatment testosterone data is unavailable, the authors may provide reasons why.

**Response**:

In this study, we could not compare the pre-treatment testosterone because most of CAB and ADT patients did not evaluate pre-treatment testosterone. Recent study demonstrated that pre-treatment androgen levels are associated with OS in mCRPC patients treated with androgen synthesis inhibitors. However, to our knowledge, there is no study which demonstrated that pre-treatment androgen levels are associated with OS in mHNPC patients. In addition, the pre-treatment testosterone data is unavailable in the LATITUDE study, either. Therefore, we did not show the baseline testosterone level in this study.
